# Editorial: Engineering the Microbial Platform for the Production of Biologics and Small-Molecule Medicines, Volume II

**DOI:** 10.3389/fmicb.2022.827181

**Published:** 2022-03-21

**Authors:** Dipesh Dhakal, Eung-Soo Kim, Mattheos Koffas

**Affiliations:** ^1^Department of Medicinal Chemistry, Center for Natural Products, Drug Discovery and Development, University of Florida, Gainesville, FL, United States; ^2^Department of Biological Engineering, Inha University, Incheon, South Korea; ^3^Department of Chemical and Biological Engineering, Rensselaer Polytechnic Institute, Troy, NY, United States

**Keywords:** metabolic engineering, *E. coli* - *Escherichia coli*, whole cell biocatalysis, antibiotics, structural modification, biotransformation

Microorganisms have been prominently used for the production of diversified small molecules to larger macromolecular complexes with wide applications in human welfare. These chemical scaffolds or biologics are employed for their application in diagnostics or ailment and treatments of different diseases (Dhakal et al., 2019). Fundamentally, the wild-type native strain or engineered native strain or engineered heterologous strains ranging from prokaryotes such as Gram negative *Escherichia coli*, Gram-positive *Streptomyces* and *Bacillus*, and eukaryotes such as yeast are used for the production of these therapeutics (Dhakal and Sohng, 2017; Ke and Yoshikuni, 2020). The papers published in this Research Topic showcase different strategies of microbial engineering for achieving these therapeutically important agents.

Ban et al. used the *E. coli* for heterologous expression of genes from *Micromonospora* and *Staphylococcus* after codon-optimization. The expressed proteins were used for *in-vitro* enzymatic reactions for synthesizing three amikacin analogs with structural modifications in the amino groups. The structure of modified compounds was confirmed by mass spectrometry and nuclear magnetic resonance (NMR) spectroscopy. These molecules were evaluated for their antibacterial activity and cytotoxicity. Among them, 6′-N-acyl-3″-N-methylated analogs showed improved antibacterial activity against the multidrug-resistant gram-negative bacteria tested. These molecules exhibited reduced *in vitro* nephrotoxicity in comparison to amikacin. This study demonstrated that the modifications of the 6′-amino group as well as the 3″-amino group have noteworthy advantages for circumventing the aminoglycoside resistance mechanism.

Heo et al. utilized the heterologous host *E. coli* for *de novo* synthesis of 11-methoxy-bisnoryangonin through *in-vivo* production approach. For this purpose, the entire biosynthetic pathway was reconfigured and optimized for obtaining the yield quantity of product starting from a simple sugar. The cDNA (*pnPKS*) of chalcone synthase (CHS)-like type III PKS, was obtained from the leaves of *Piper nigrum*. The PnPKS protein was incubated with ferulic acid whereas the enzyme catalyzed lactonization instead of chalcone or stilbene formation. The new product was characterized as a styrylpyrone, 11-methoxy-bisnoryangonin. Furthermore, an artificial biosynthetic pathway was reconstructed which contained ferulic acid biosynthetic genes: *optal, sam5, com*, and *4cl2nt*, along with the *pnPKS*. The engineered L-tyrosine overproducing *E. coli* ΔCOS1 strain was transformed with these five biosynthetic genes and cultured for 24 h in a minimal glucose medium. The final yield of 11-methoxy-bisnoryangonin production was ~52.8 mg/L, which is ~8.5-fold higher than that in the parental *E. coli* strain.

Yan et al. used a directed evolution and host engineering approach in *E. coli* to improve the production of pterostilbene. First, the heterologous biosynthetic pathway of pterostilbene, including tyrosine ammonia lyase, p-coumarate: CoA ligase, stilbene synthase, and resveratrol O-methyltransferase, were successively generated by the error-prone PCR. The genetic circuit containing the engineered enzymes with higher biocatalytic efficiency elevated the pterostilbene production by 13.7-fold. Then, a biosensor-guided genome shuffling strategy was used to improve the availability of the precursor, L-tyrosine. *E. coli* TYR-30 was used to produce pterostilbene, whereas the shuffled strain produced 80.04 ± 5.58 mg/L pterostilbene which is ~2.3-fold greater than the highest titer reported to date.

Lu et al. utilized the whole cell biocatalysis by two fungal strains, *Aspergillus awamori* and *Trichoderma reesei* to process and convert arctiin from *Fructus arctii* powder into arctigenin. They developed an optimized fermentation process by adjusting the carbon and nitrogen source/ratio, fermentation time, pH, liquid volume, inoculation volume, and substrate solid-liquid ratio. This resulted in an arctiin conversion rate of 99.84%, and the dissolution rate of the final product was 95.74%, with a loss rate as low as 4.26%. After the fermentation of *Fructus arctii* powder, the average yield of arctigenin was ~19.51 mg/g.

These papers fundamentally illustrate the applicability of different microbial platforms such as *E. coli* and fungi for generating products of interest. Recently, the availability of robust techniques for genome sequencing has assisted in exploring the possibility of unique and signature enzymes. In addition, the genome guided application of advanced tools for generating artificial genetic circuits/metabolic pathways, or multiplexed genome engineering utilizing CRISPR has advanced the engineering approaches to the next level. Further developments in computational approaches such as artificial intelligence (AI) and machine learning approaches (MLA) has a significant impact on fine-tuning the production profiles by targeted protein level engineering or holistic microbial engineering by reconfiguring the precursor pathways, regulation mechanism, and overall metabolic flux.

## Author Contributions

DD wrote the manuscript. E-SK and MK revised and corrected the manuscript. The final draft of the manuscript was finalized and approved for publication by all the authors.

## Conflict of Interest

The authors declare that the research was conducted in the absence of any commercial or financial relationships that could be construed as a potential conflict of interest.

## Publisher's Note

All claims expressed in this article are solely those of the authors and do not necessarily represent those of their affiliated organizations, or those of the publisher, the editors and the reviewers. Any product that may be evaluated in this article, or claim that may be made by its manufacturer, is not guaranteed or endorsed by the publisher.
